# Good things come to those who mate: analysis of the mating behaviour in the menstruating rodent, *Acomys cahirinus*

**DOI:** 10.1186/s40850-022-00112-1

**Published:** 2022-02-25

**Authors:** Jarrod McKenna, Nadia Bellofiore, Peter Temple-Smith

**Affiliations:** 1 Department of Obstetrics & Gynaecology, School of Clinical Sciences, Education Program in Reproduction & Development, Monash University, Clayton, VIC Australia; 2 The Ritchie Centre, Hudson Institute of Medical Research, Clayton, VIC Australia

**Keywords:** *Acomys cahirinus*, Copulation, Ejaculation, Intromission, Menstruation, Spiny mouse

## Abstract

**Background:**

The Egyptian spiny mouse (*Acomys cahirinus*) is the only known rodent to exhibit true, human-like menstruation and postpartum ovulation, and is an important new model for reproductive studies. Spiny mice do not produce a visible copulatory plug, and calculation of gestational age is therefore restricted by the need to use mated postpartum dams. The current inefficient method of monitoring until parturition to provide a subsequent estimate of gestational age increases study duration and costs. This study addressed this issue by comparing the mating behaviour of spiny mice across the menstrual cycle and proposes a more accurate method for staging and pairing animals that provides reliable estimates of gestational age. In experiment 1, mating behaviour was recorded overnight to collect data on mounting, intromission, and ejaculation (*n* = 5 pairs per stage) in spiny mice paired at menses and at early and late follicular and luteal phases of the menstrual cycle. In experiment 2, female spiny mice were paired at the follicular or luteal phases of the menstrual cycle to determine any effect on the pairing-birth interval (*n* = 10 pairs).

**Results:**

We report a broad mating window of ~ 3 days during the follicular phase and early luteal phase of spiny mice. Males displayed a discrete ‘foot twitch’ behaviour during intromission and a brief copulatory lock during ejaculation. Litters were delivered after 40–43 days if pairing occurred during the mating window, compared with 46–48 days for spiny mice paired in the late luteal phase. When pairing occurred during the late luteal phase or menses no mating activity was observed during the recording period.

**Conclusion:**

This study clearly demonstrates an effect of the menstrual cycle on mating behaviour and pregnancy in the spiny mouse and provides a reliable and more effective protocol for estimating gestational age without the need for postpartum dams.

**Supplementary Information:**

The online version contains supplementary material available at 10.1186/s40850-022-00112-1.

## Background

Copulatory behaviour in rodents and most other mammals is tightly linked to their reproductive physiology, behavioural estrus, and the timing of ovulation [1–3]. This relationship is particularly useful in gestational studies in laboratory rodents where copulation and pregnancy are highly predictable and can be confirmed non-invasively. The Egyptian spiny mouse, *Acomys cahirinus,* is a rodent native to North Africa that has recently been used as a laboratory rodent in biomedical research. While limited information is available regarding *A. cahirinus* reproductive biology, recent research has highlighted several reproductive characteristics that are rare in rodents. *Acomys cahirinus* produce small litters with precocial young [4] and a relatively long gestation (~ 39 days; [5]), cannot become pseudopregnant [6], and, more recently, were the first rodents to exhibit a natural, human-like menstrual cycle [7] and a menopause-like transition [8]. Interestingly, unlike most rodents, *A. cahirinus* also do not produce a visible copulatory/seminal plug [9], which provides significant challenges when using mated females for gestational studies as ejaculation, and therefore early pregnancy, cannot be confirmed non-invasively.

However, based on observations in our breeding colony, female spiny mice also experience a postpartum ovulation, where ovulation and mating occur within 48 h of parturition [10]. Using this knowledge, estimates of gestational age are currently calculated from the date of delivery of the previous term litter [11, 12]. While this method provides a useful and relatively accurate estimate of gestational age, it is time-consuming, costly, and inefficient. It requires the monitoring of breeder pairs until parturition (a minimum of 40 days from pairing virgin females and no guarantee that females will have mated immediately upon pairing) plus the subsequent timing of pregnancy to the required stage of gestation.

Observations from our laboratory have also identified variable pairing-to-birth intervals that ranged from 40–46 days from when females were paired at unknown stages of the 9-day menstrual cycle. This suggested that stage of the menstrual cycle influences mating behaviour that results in these variations in gestational outcomes in *A. cahirinus*. Although copulatory behaviour in this species has been described [13], in a study that also preceded the discovery of menstruation in *A. cahirinus*, no attempt was made to correlate the phase of the reproductive cycle with the timing of copulation and birth. In this study, the data recording methods used were not clearly detailed, and no video or photographic evidence of mating behaviour was provided. With the current access to improved recording technologies, a more accurate and effective method for timing mating, insemination and pregnancy can now be achieved. In this study, we have built upon the previous study by investigating the effect of menstrual cycle stage on receptivity, copulation, and birth outcomes in the Egyptian spiny mouse using digital recording equipment and used these data to propose a more effective method for estimating gestational age in the spiny mouse.

## Results

### Experiment 1

**Fig. 1:**
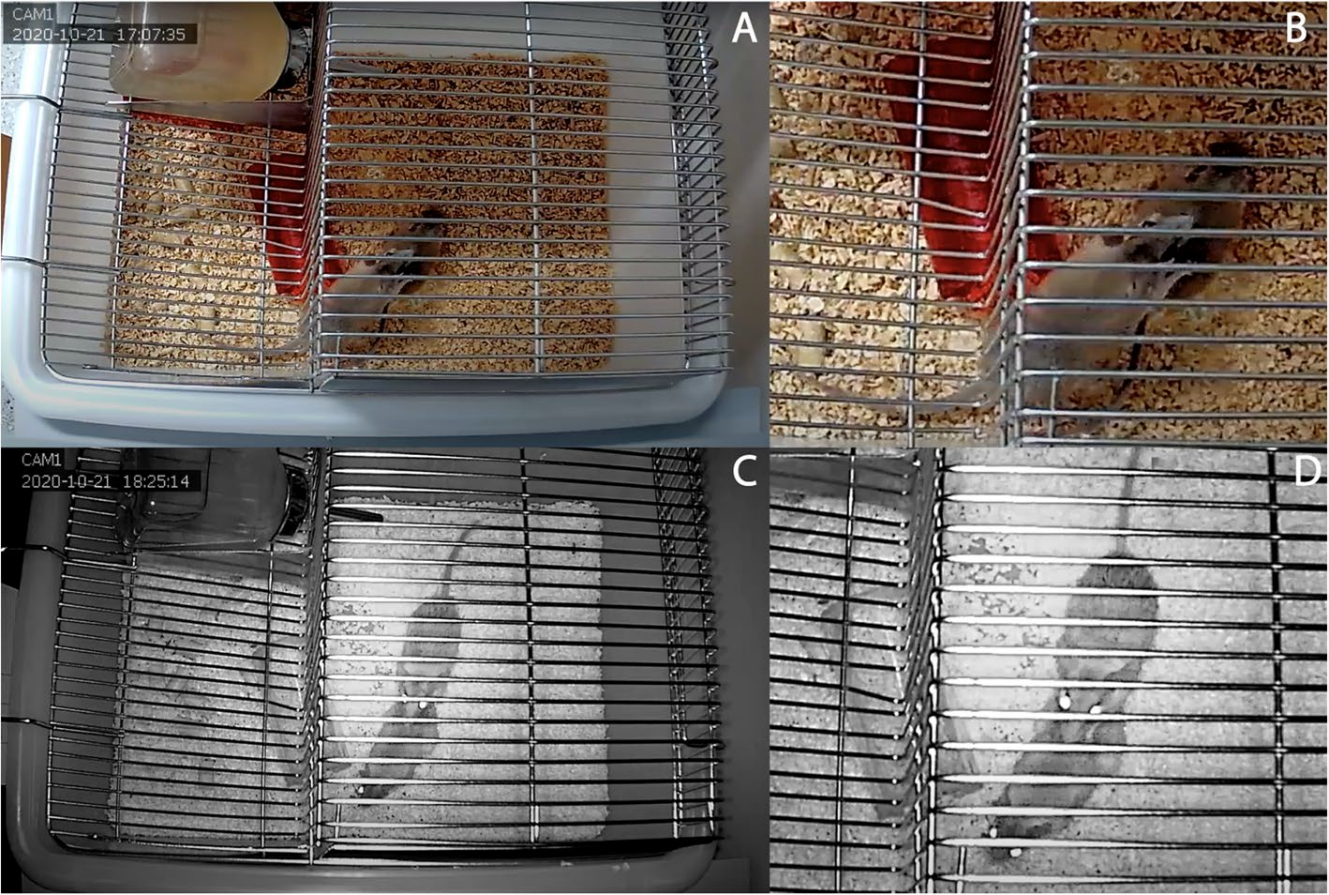
Mounting behaviour during the lights-on (**A**, **B**) and lights-off (**C**, **D**) periods in spiny mice. Male spiny mice approach females from behind and place front paws on the middle of the female’s back. Females will either allow mating to occur or move to prevent unwanted mating. Frames 2B and 2D are zoomed in images (2X) of 2A and 2C respectively

Male spiny mice approached females from behind prior to mounting (Fig. 1, Supplementary videos 1–2) and females either moved away, sometimes climbing the wire cage top to prevent unwanted advances or were receptive and allowed the male to mate with her.

**Fig. 2:**
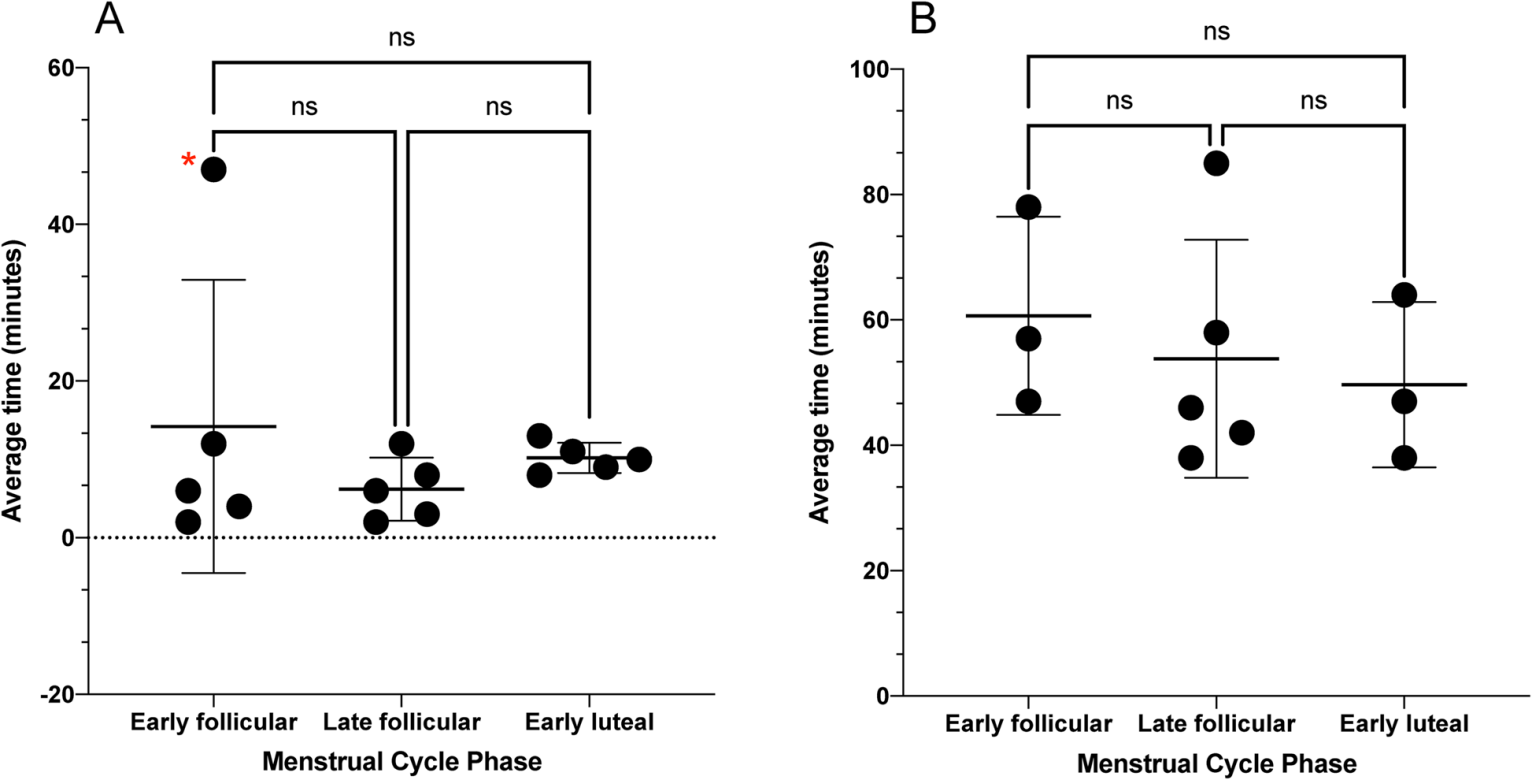
Mount latency (**A**; ML) and intromission latency (**B**; IL) in spiny mice paired at different stages of the menstrual cycle. ML and IL are similar between early follicular, late follicular and early luteal phases. Intromission events were seen in 3/5 pairs during the early follicular and early luteal phases, and in 5/5 pairs during the late follicular phase. All data are mean ± SD, significance set at *p* < 0.05 and * reflect outliers within cycle stages. ANOVA for ML: F_3,12_ = 0.6485, *P* = 0.5402. ANOVA for IL: F_2,8_ = 0.3259, *P* = 0.7310

Mounting was usually initiated rapidly after introduction and was defined as the male clasping the female’s back with his forepaws. Mount latencies were similar between the early follicular, late follicular, and early luteal phase (*p* > 0.05), and generally under 10 min (Fig. 2A). However, during the late luteal phase mounting was only observed in one of the five pairs and occurred 85 min after introduction. No additional copulatory behaviours were seen in this pair, and no copulatory behaviours were seen when pairing occurred during menses in all pairs.

Intromission was a discrete event in which the female stood still while the male climbed further along her back with no apparent biting of the female’s back or nape. Males also display a clear, and previously unrecorded, foot twitch during the brief period of intromission (~ 1 s; Supplementary videos 1–2) followed by a ballistic dismount.

**Fig. 3:**
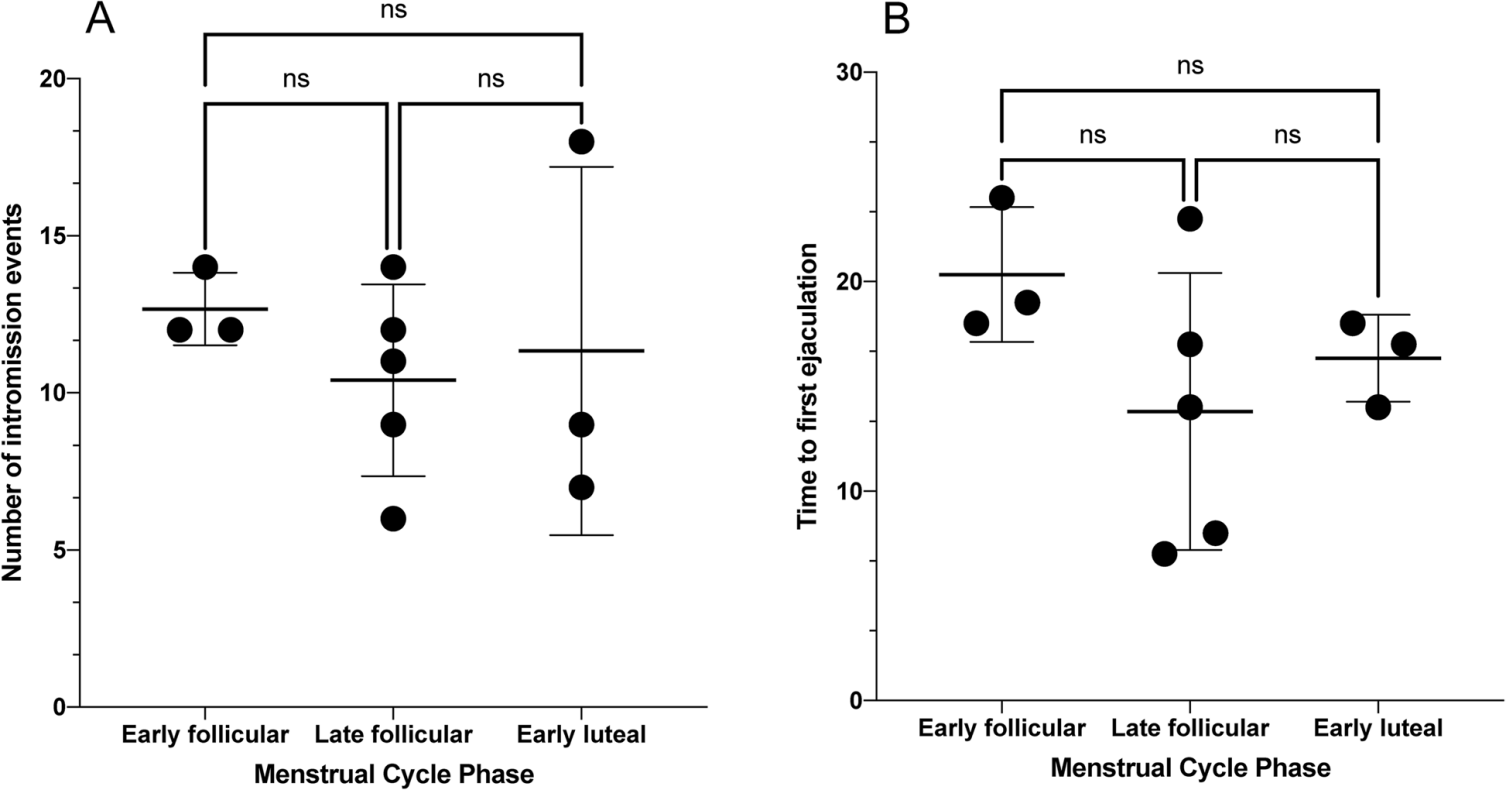
Intromission frequency (**A**; IF) and ejaculation latency (**B**; EJL) in spiny mice paired at different stages of the menstrual cycle. IF and EJL are similar between those cycle stages where intromission occurred. Intromission occurred in a series between 6 and 18 events (**A**) and EJL occurred between 7 and 24 min after the first intromission (**B**). All data are mean ± SD and significance set at *p* < 0.05. ANOVA for IF: F_2,8_ = 0.3556, *P* = 0.7113. ANOVA for EJL: F_2,8_ = 1.569, *P* = 0.2662

Intromission was seen in all (5/5) late follicular phase pairings, but only in 3 each of the 5 early follicular and 5 luteal phase pairings (Fig. 2B). Intromission latencies were similar between the early follicular, late follicular, and early luteal phases (Fig. 2B, 3A; *p* > 0.05), and the first intromission typically occurred approximately one hour after introduction (Fig. 2B; *p* > 0.05). Similarly, number of intromissions were also similar across these groups and generally occurred in a series of between 6 and 18 intromissions (11.3 ± 3.4 SD; Fig. 3A; *p* > 0.05). All pairings in which intromission was seen also resulted in ejaculation, which was confirmed by the presence of spermatozoa in the vaginal lavage. There were no behavioural signs that intromission or, by its association, ejaculation had occurred in any of the late luteal or menses phase pairs. Intromissions were confirmed as non-ejaculatory as females smeared after each intromission event contained no spermatozoa.

Ejaculation was distinguished from intromission by the difficulty of the mating pair to separate (Supplementary videos 3–4) following a discrete, but extremely brief, copulatory lock (~ 1 s). Copulatory locks were also confirmed to be ejaculatory events by the presence of spermatozoa in vaginal smears of females immediately following locking events (Supplementary Fig. 1). In all 3 phases of the menstrual cycle where mating was successful, ejaculation occurred after a similar interval of between 7 and 24 min (16.3 ± 5.3 SD) after the first intromission (Fig. 3; *p* > 0.05) and always followed a series of non-ejaculatory intromissions.

**Fig. 4:**
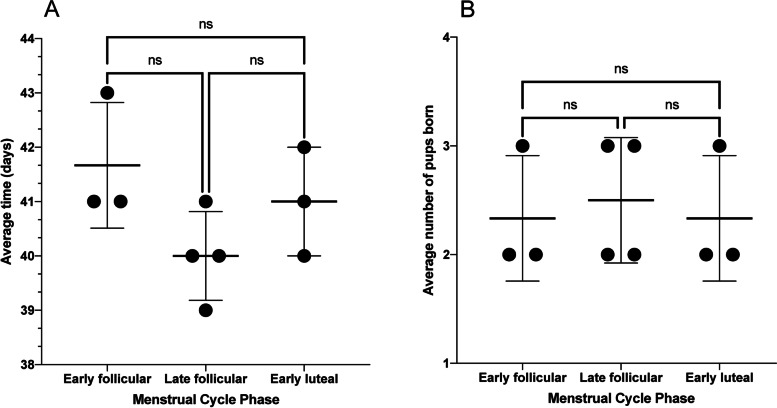
Pairing to birth interval (**A**) and number of pups born (**B**) in spiny mice paired at different stages of the menstrual cycle. Average time to litter down and number of pups born were similar across all cycle stages where ejaculation was seen. Data are mean ± SD and statistical significance set at *p* < 0.05. ANOVA for birth interval: F_2,7_ = 2.590, *P* = 0.1439. ANOVA for number of pups: F_2,7_ = 0.1000, *P* = 0.9061

Most pairings resulted in a single ejaculation (8/11) during the observational period, however, based on observed locking events, three males ejaculated twice (2 in late follicular, 1 in early luteal) with a mean interval between ejaculations of 36 ± 11 min. No visible pelvic thrusting was evident during either intromission or ejaculation and genital grooming followed all intromission and ejaculation sequences. In all pairings for which ejaculation was observed, vaginal lavages from the following morning revealed no spermatozoa or visible seminal plugs. Also, litters in this experiment were born with a mean pairing to birth interval of 40.9 ± 0.8 days and an average litter size of 2.4 ± 0.1 pups (Fig. 4A and B).

### Experiment 2

**Fig. 5:**
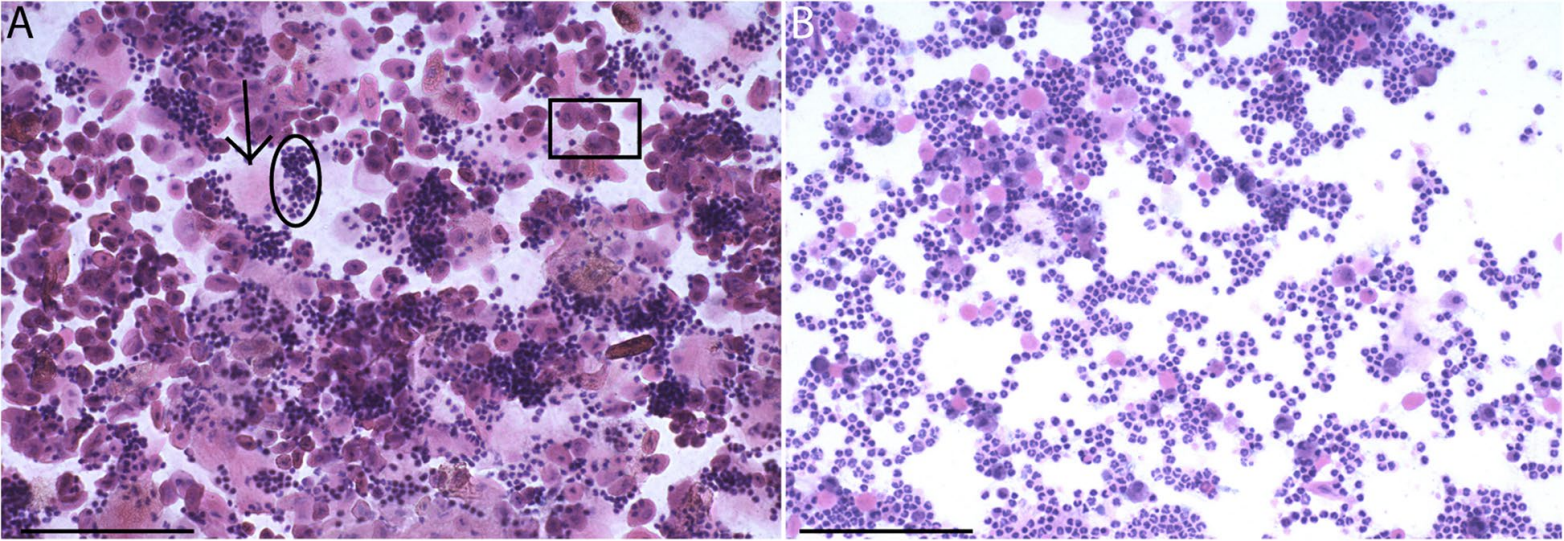
Comparative vaginal cytology from the early (**A**) and late (**B**) luteal phase. The early luteal phase cytology contained a mixture of cornified epithelial cells (CECs; arrow), leukocytes (circle) and nucleated epithelial cells (NECs; square). Late luteal phase cytology was dominated by leukocytes with a comparatively smaller number of CECs and no NECs. Scale bars = 100um

In experiment 2, only 4/5 females paired in the follicular phase and 1/5 paired in the luteal phase gave birth within 45 days of pairing. The female paired in the luteal phase which gave birth before 45 days had vaginal cytology containing a mixture of cornified epithelial cells (CECs), leukocytes and nucleated epithelial cells (NECs) (Fig. 5A) that was more typical of an early luteal phase vaginal smear in *A. cahirinus*. The remaining 4 females from the luteal phase pairings all showed vaginal cytology typical of the late luteal phase at pairing (Fig. 5B).

Litters were born between 39 and 43 days after pairing between the follicular and early luteal phase (mean 41.7 ± 1.2 SD; *p* > 0.05) by all females paired in the follicular and early luteal phases. In contrast, the pair-birth interval in 4/5 females paired in the late luteal phase was significantly longer (46–48 days after pairing, mean 46.6 ± 0.9 SD; *p* < 0.05), and the remaining female did not give birth.

## Discussion

This study has described an improved analysis of spiny mouse copulatory behaviour from that published by Dewsbury and Hodges [13] and provided new data on the relationship between mating success and the spiny mouse menstrual cycle in our captive colony. In their original study, Dewsbury and Hodges argued that male copulatory patterns cannot be predicted from knowledge of the female estrous cycle. Our study challenges this assertion. We have demonstrated the clear presence of a broad ‘mating window’ in which mating can occur during the early follicular, late follicular, and early luteal phase phases of the menstrual cycle, but not during the late luteal or menses phases. In mammals, gonadal steroids are known to affect sexual behaviour and mating receptivity [14]. This relationship is true in female rodents [15], and also women, where copulation is significantly more frequent during their ovulatory window [16, 17] when testosterone and dihydrotestosterone (DHT) concentrations are the highest [18]. Although androgen concentrations during the menstrual cycle in *A. cahirinus* have not been reported, our observations that females may also be receptive to mating several days either side of ovulation suggest a possible relationship between copulation frequency and changing androgen levels.

Unexpectedly, neither the pair-birth interval nor the number of pups born were significantly different across cycle phases where pups were born. This is in contrast to hamsters and mice where a significant difference in litter size is attributed to timing of mating [19, 20]. While time between the early follicular and early luteal phases of the menstrual cycle may differ by up to 2 days in the spiny mouse [7], it appears this time may be too little to observe a noticeable difference in litter size and pair-birth intervals. Further, a strong inverse relationship between litter size and gestation length has been reported in mice, rats, and gerbils [20–22]. However, differences are only apparent when comparing higher- and lower-order pregnancies in these species. Given the naturally small litter sizes in spiny mice (1–5; [4]) and higher-order pregnancies generally being born to multiparous dams (unpublished data from our colony), this may explain the similar pair-birth intervals we have observed.

Moreover, individual pairs established during the early follicular to early luteal phases of the cycle produce litters after a similar period to those reported in postpartum female spiny mice (41 days ± 4.4 days (SD); unpublished data from our colony). While litters were born > 45 days post-pairing in luteal phase females, these are most likely resultant of mating and pregnancies from the subsequent cycle, rather than the paired cycle. Thus, our method of timing pairing to a particular phase of the menstrual cycle, as outlined here, resulted in similar gestational outcomes to mated postpartum dams, while reducing study duration and costs.

Another distinction between our study and that of Dewsbury and Hodges [13], is the use of vaginal lavage to confirm behavioural cues for, and the timing and location of, ejaculation within the female reproductive tract. No spermatozoa were present in vaginal lavages taken immediately after individual, non-locking, intromission events. Despite interruptions to mating activities to obtain vaginal smears, and the potential stress involved in this procedure, spiny mice pairs rapidly resumed sexual activity after each consecutive vaginal lavage. This is an interesting and important observation for future mating studies because, although spiny mice are susceptible to stress (unpublished data from our colony), it appears that the mating drive is strong enough to overcome any stress caused by the disturbance of removing females briefly for vaginal lavage. Spermatozoa were seen in vaginal smears of females immediately after a copulatory lock, suggesting intravaginal ejaculation. However, as post-coital reproductive tracts of females were not examined in this study, we cannot rule out the possibility of intrauterine or intracervical insemination, as occurs in some other species like pigs and the camelids [23].

Our mating behavioural analysis reveals several similarities to Dewsbury and Hodges [13]. We observed no pelvic thrusting during either intromission or immediately prior to ejaculation, and a series of intromissions always preceding ejaculation. We also observed no obvious lordosis in female spiny mice during intromission or ejaculation; a feature that is typical of murid copulation [24]. Instead, we observed a distinct male foot twitch behaviour, which was not reported by Dewsbury and Hodges [13]. Interestingly, a similar behaviour, ‘thumping’, was reported in Mongolian gerbils in which either individual taps its hind feet against the cage floor immediately following coitus [25]. However, the cause of this behaviour is ambiguous as it presents in both sexes in other non-coital settings and has been considered a sign of stress [26]. In contrast, the male spiny mouse foot twitch that occurred only during coitus, we interpret as a behavioural response of males to pre-ejaculatory penile insertion during mounting on a female not presenting any clear lordosis. While multiple intromissions have been suggested as a necessary requirement to trigger ovulation in several rodent species [1, 27], *A. cahirinus* present with spontaneous, rather than induced ovulation [6, 7]. Therefore, the multiple intromissions observed here are more likely a prerequisite to stimulate ejaculation, rather than to stimulate ovulation in *A. cahirinus*.

We also confirm the presence of a copulatory lock and no obvious copulatory plug in *A. cahirinus*. In an extensive review of 118 mammalian species, Dewsbury [1] categorised mating behaviour into 16 categories; the most common patterns being # 9 (no lock, intravaginal thrusting, multiple intromissions and ejaculations) and #13 (no lock, no intravaginal thrusting, multiple intromissions and ejaculations). Interestingly, all species in these two categories were either primates or rodents, with most rodents falling into category #13. From this, it appears that mating behaviour in *A. cahirinus* is broadly similar to other rodent species but with the addition of a copulatory lock. Seminal plugs are common in rats and guinea-pigs, but only observed in a few mouse species [15], and these non-plugging species generally have copulatory locks and reduced or underdeveloped accessory glands (reviewed by [28]). Further, Voss [15] argued that if there is a causal relationship between the presence of copulatory locks and the absence of plug formation, they ‘must serve much the same function(s) as the plugs they presumably replaced’.

Male spiny mice have a normal complement of accessory glands typical of many murid rodents [29], with a large well-developed seminal vesicle but a comparatively small prostate and coagulating glands. Hartung and Dewsbury [28] have suggested that well-developed accessory glands are required for rodent seminal plug formation, but no copulatory plug has been observed in spiny mice. However, coagulation studies using spiny mouse accessory gland secretions [29], especially mixing of extracts from the seminal vesicles and the coagulating glands, shows coagulum formation. Together, this suggests the possibility of a covert post-ejaculatory seminal plug in spiny mice, perhaps deep within the vagina against the cervix or within the cervical canal.

The functional role of copulatory plugs in rodents has been debated for centuries [30] and several hypothesis have been suggested. These include prevention of insemination by rival males, assisting sperm transport, induction of pseudopregnancy and prevention of sperm leakage from the vagina [15]. None of these hypothesis are likely to apply in *A. cahirinus* considering the extremely brief lock compared to true locking species [31], and the demonstrated inability to induce pseudopregnancy in this species [6]. An alternative explanation is that, despite the brief ejaculatory lock, spiny mice deposit most or all of the ejaculate directly into the cervix or uterus like camelids and pigs [23]. Although spermatozoa were seen in vaginal lavages immediately following the brief locking events, these spermatozoa may be flowback through the cervix or leakage from the penis during withdrawal from the vagina. Considering this, if ejaculation does occur within the cervix or the uterus, formation of a small, very temporary, seminal plug may assist in maintaining spermatozoa at the site of ejaculation. Future studies of female spiny mouse reproductive tracts following coitus may provide answers to these questions on the site of insemination and presence of post-coital seminal plug, and provide new information on sperm concentration, survival, and transit through the female tract.

## Conclusion

This study has extended and improved the analysis of spiny mouse mating behaviour reported by Dewsbury and Hodges [13] and, importantly, it provides a comprehensive description of mating behaviour across the recently discovered menstrual cycle of this species. We have defined a more reliable and efficient method for staging gestational age in female spiny mice by describing a discrete mating window where mating behaviour and pairing-birth intervals are highly predictable. Ejaculation was seen in 11/15 pairs (73%) during the mating window, and an average of 2 pups were delivered from 10/11 (91%) pairs between 38 and 43 days later. Moreover, females paired during the late luteal or menses phases were not receptive to mating, and males displayed no mating behaviour during these phases. This study has confirmed its original aim of providing a method for more reliably estimating gestational and fetal age in pregnant spiny mice and will improve gestational studies by reducing both the research time and financial costs of using postpartum animals.

## Methods

### Ethics approval and animal use

All experimental procedures carried out in this study were performed according to the ARRIVE guidelines, and adhered to the Australian Code of Practice for the Care and Use of Animals for Scientific Purposes. All animals (*n* = 70) were sourced from our breeding colony and approved for use by the Monash Medical Centre Animal Ethics Committee (MMCB 2019/13BC). Sexually mature spiny mice (3 – 9 months of age) were housed in sex-segregated groups up to nine per cage or as breeding pairs under a 12:12 h light:dark cycle at 25–28 °C and humidity of 30–40% [4]. Cages were lined with wood shavings and plastic tunnels, cardboard boxes or tissue paper provided as environmental enrichment. Food (rat and mouse cubes; Specialty Feeds, Glen Forest WA) and water was provided ad libitum, with weekly supplements of fresh carrots and celery.

### Experimental design

Two experiments were conducted in this study. The first experiment was designed to record and characterise the mating behaviour of spiny mice at different stages of the menstrual cycle. The second experiment was used to determine the effect of menstrual cycle stage on spiny mouse pairing-birth intervals.

### Experiment 1 (*n* = 25 pairs; 50 animals)

**Fig. 6:**
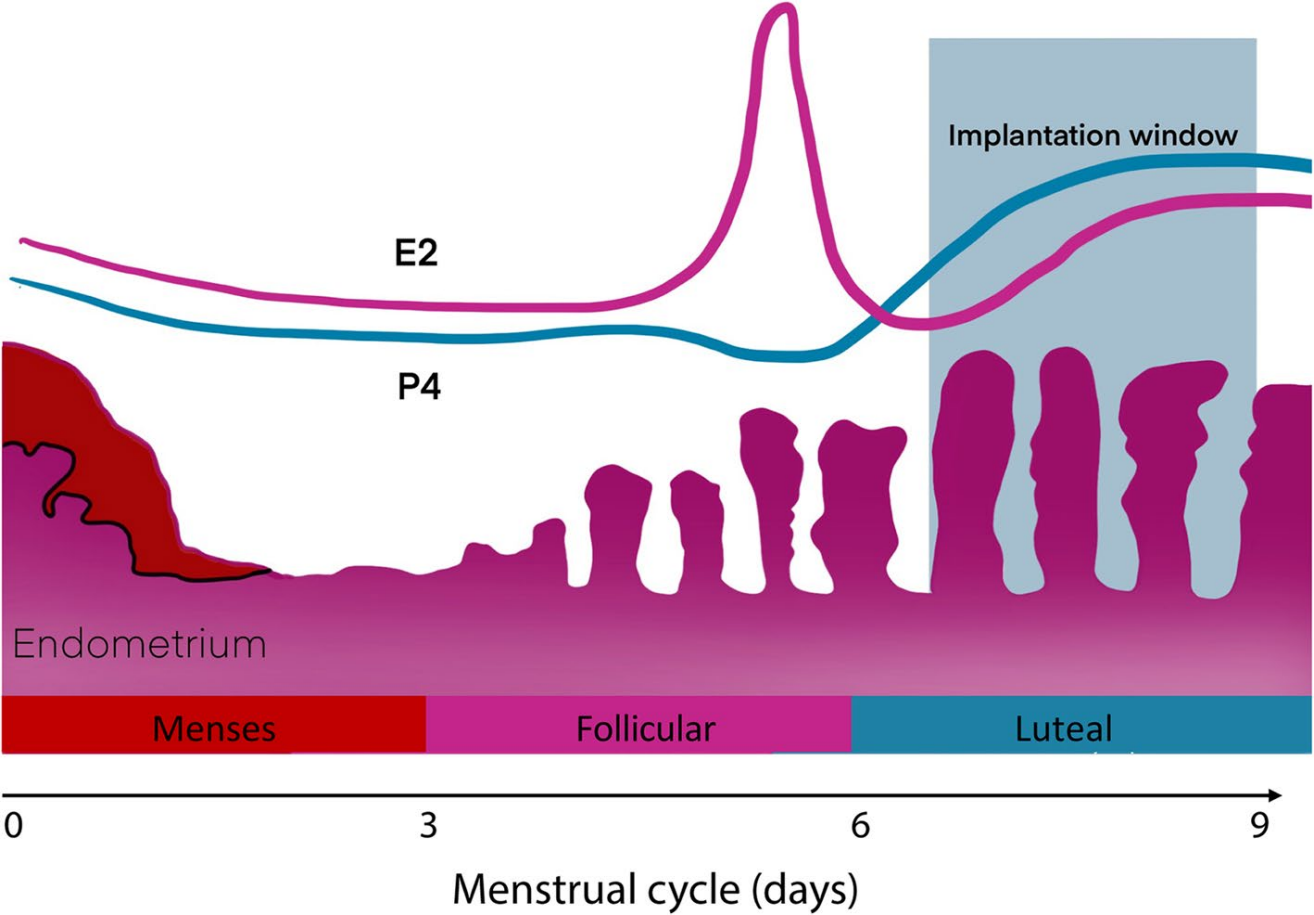
Stages of the Spiny Mouse Menstrual cycle. Menses in *A. Cahirinus* lasts approximately 3 days and is followed by the follicular phase where several follicles are matured under the influence of estradiol (**E2**). Following a surge of **E2**, the dominant follicle ruptures releasing the mature oocyte, and subsequently forms a progesterone (**P4**) secreting corpus luteum. Following ovulation, the luteal phase begins, which encompasses the implantation window whereby a healthy blastocysts implants into a receptive uterus to establish pregnancy

The aim of this experiment was to compare behavioural cues for copulation with those outlined by Dewsbury and Hodges [13], and in particular, mounting, intromission and ejaculation. Spiny mice (*n* = 5 per phase) were paired at the early follicular (day 3–4), late follicular (day 4–5), early luteal (day 5–7), late luteal (day 7–9) and menses (days 1–3) phases of the cycle (Fig. 6). Being a naturally crepuscular species [32], the mating activity of each pair was recorded during the night for at least 6 h, commencing at 5 pm and ending the following morning at 8am.

Males and females were separated and returned to their respective home cages the following morning and females were lavaged vaginally on the morning of separation to check for signs of semen or a copulatory plug. Each female was then monitored for 30 days after pairing, when females were visibly pregnant and pups were palpable (~ 30 days; unpublished data from our colony). Females that were visibly pregnant were removed from their home cages and housed in a single cage to litter down; those that were not pregnant were left undisturbed in their home cage.

### Recording

**Fig. 7:**
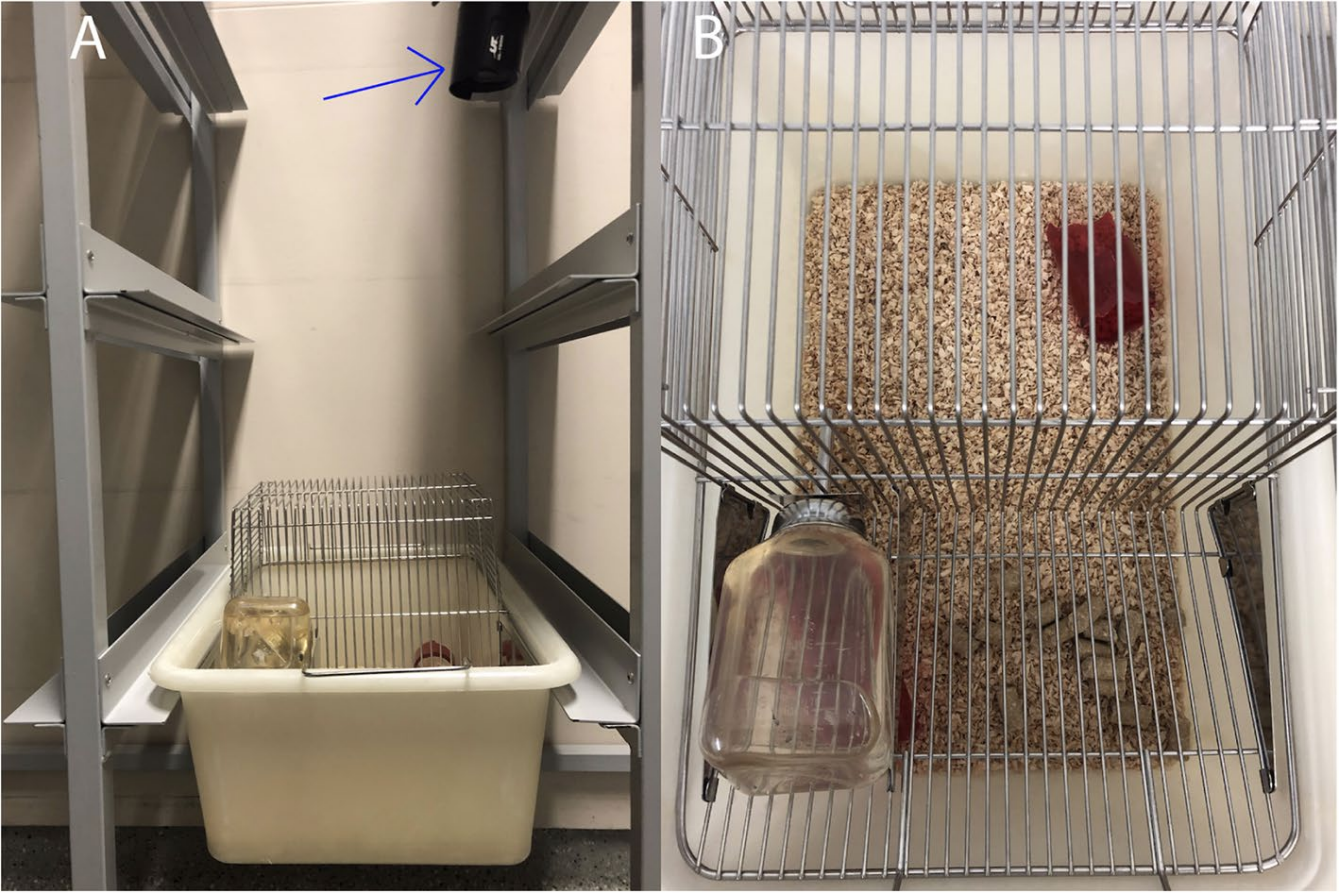
Recording setup used for recording spiny mouse mating behaviour. Cameras (arrow) are attached to the cage racks and placed two shelves above the breeder cage (**A**). Water is provided on top of the cage lid, whilst food and enrichment devices are provided within the cage (**B**)

Mating behaviour was recorded in a clean cage, with fresh bedding and enrichment (as required by ethics), using a 1080p wireless security camera system (cctv-wf-cla-4c-4b; UL-Tech, Australia) (Fig. 7). At least 6 h of footage was examined after pairing.

### Mating behaviour

Mating behaviour was measured as described by Dewsbury and Hodges [13] with time to live birth (pairing-birth interval) and number of pups born as additional measures. The following behaviours were included in the behavioural assessment: mount latency (ML; time from pairing to first mount), intromission latency (IL; time from pairing to first intromission), intromission frequency (IF; number of intromissions prior to ejaculation), ejaculation latency (EJL; time from first intromission to ejaculation), ejaculation interval (EJI; time from ejaculation to the next intromission) and ejaculation frequency (EJF; number of ejaculations observed). Females (*n* = 1 pair from each cycle stage) were also subjected to vaginal lavage immediately after copulatory behaviours were observed. These behaviours were considered to be ejaculatory events and vaginal lavages were performed to determine if spermatozoa was present in the vagina to confirm ejaculation.

### Experiment 2 (*n* = 10 pairs; 20 animals)

The aim of this experiment was to determine the pair-birth interval of animals paired in the follicular and luteal phase when animals are left together until pregnancy was visible, rather than removed the following morning of pairing. Stage of the menstrual cycle was determined [7] in sexually mature, cycling females (*n* = 5 pairs per phase) prior to pairing during the follicular or luteal phases (Fig. 6) of the menstrual cycle. Animals in this experiment were paired at 1700 h and left together until there were visible signs of pregnancy as described in experiment 1.

### Statistical analysis

All data were analysed using Prism 8 software (GraphPad). Data from experiment 1 were tested for normality using the Shapiro–Wilk test before further analysis. One-way analysis of variance (ANOVA) was used to compare data between cycle phases, Tukey’s multiple comparison test was used as post-hoc analysis, and Grubb’s test was used to identify any outliers. Data from experiment 2 were subject to an unpaired t-test, and all data were reported as mean ± standard deviation (SD) and statistical significance set at *p* < 0.05.

## Supplementary Information

**Additional file 1: Supplementary figure 1.** Spermatozoa in a vaginal lavage sample following a locking event. Spermatozoa (arrows) and cornified epithelial cells (circles) in the vaginal lavage from a female spiny mouse immediately following an ejaculatory locking event. Scale bar = 100um (40X) and inset square shows spermatozoa and cornified epithelial cells at higher magnification (100X).

**Additional file 2: Supplementary video 1.** Mounting and intromission behaviours of spiny mice during the lights-on period. Male spiny mice approach females from behind prior to mounting where the male clasps the female’s flanks with his front paws. Males then display a distinct foot-twitch behaviour during intromission followed by a ballistic dismount.

**Additional file 3: Supplementary video 2.** Mounting and intromission behaviours of spiny mice during the lights-off period. Male spiny mice approach females from behind prior to mounting where the male clasps the female’s flanks with his front paws. Males then display a distinct foot-twitch behaviour during intromission followed by a ballistic dismount.

**Additional file 4: Supplementary video 3.** Locking/ejaculatory behaviours of spiny mice during the lights-on period. Spiny mouse ejaculatory behaviour is distinct from intromission by the apparent difficulty to separate, due to a brief (~1 second) copulatory lock.

**Additional file 5: Supplementary video 4.** Locking/ejaculatory behaviours of spiny mice during the lights-off period. Spiny mouse ejaculatory behaviour is distinct from intromission by the apparent difficulty to separate, due to a brief (~1 second) copulatory lock.

## Data Availability

The datasets used and/or analysed during the current study are available from the corresponding author on reasonable request.

## Abbreviations

CECs: Cornified epithelial cells
NECs: Nucleated epithelial cells
ML: Mount latency
IL: Intromission latency
IF: Intromission frequency
EJL: Ejaculation latency
EJI: Ejaculation interval
EJF: Ejaculation frequency
DHT: Dihydrotestosterone

## References

[1] Dewsbury DA (1975). Diversity and adaptation in rodent copulatory behavior. Science.

[2] Romano JE, Keisler DH, Amstalden M (2018). Effect of copulation on estrus duration, LH response, and ovulation in Boer goats. Theriogenology.

[3] Jorge-Neto PN, Luczinski TC, de Araújo GR, Júnior JAS, de Souza TA, Dos Santos JAM (2020). Can jaguar (Panthera onca) ovulate without copulation?. Theriogenology.

[4] Dickinson H, Walker D (2007). Managing a colony of spiny mice (Acomys cahirinus) for perinatal research. Australian and New Zealand Council for the Care of Animals in Research and Training (ANZCCART) News.

[5] Brunjes PC (1990). The precocial mouse, Acomys cahirinus. Psychobiology.

[6] Bellofiore N, Ellery SJ, Temple-Smith P, Evans J (2020). Pseudopregnancy and reproductive cycle synchronisation cannot be induced using conventional methods in the spiny mouse (Acomys cahirinus). Reprod Fertil Dev.

[7] Bellofiore N, Ellery SJ, Mamrot J, Walker D, Temple-Smith P, Dickinson H (2017). First evidence of a menstruating rodent: the spiny mouse (Acomys cahirinus). Am J Obstet Gynecol..

[8] Bellofiore N, George E, Vollenhoven B, Temple-Smith P (2021). Reproductive aging and menopause-like transition in the menstruating spiny mouse (Acomys cahirinus). Human Reproduction.

[9] Haughton CL, Gawriluk TR, Seifert AW (2016). The biology and husbandry of the African spiny mouse (Acomys cahirinus) and the research uses of a laboratory colony. J Am Assoc Lab Anim Sci.

[10] McKenna J, Bellofiore N, Dimitriadis E, Temple-Smith P (2021). Postpartum ovulation and early pregnancy in the menstruating spiny mouse. Acomys cahirinus Scientific reports.

[11] O'Connell BA, Moritz KM, Walker DW, Dickinson H (2013). Sexually dimorphic placental development throughout gestation in the spiny mouse (Acomys cahirinus). Placenta.

[12] Ireland Z, Castillo-Melendez M, Dickinson H, Snow R, Walker D (2011). A maternal diet supplemented with creatine from mid-pregnancy protects the newborn spiny mouse brain from birth hypoxia. Neuroscience.

[13] Dewsbury DA, Hodges AW (1987). Copulatory behavior and related phenomena in spiny mice (Acomys cahirinus) and hopping mice (Notomys alexis). J Mammal.

[14] Jennings KJ, de Lecea L (2020). Neural and Hormonal Control of Sexual Behavior. Endocrinology.

[15] Voss R (1979). Male accessory glands and the evolution of copulatory plugs in rodents.

[16] Wilcox A, Day Baird D, Dunson DB, McConnaughey DR, Kesner JS, Weinberg CR (2004). On the frequency of intercourse around ovulation: evidence for biological influences. Hum Reprod.

[17] Wilcox AJ, Weinberg CR, Baird DD (1995). Timing of sexual intercourse in relation to ovulation—effects on the probability of conception, survival of the pregnancy, and sex of the baby. N Engl J Med.

[18] Rothman MS, Carlson NE, Xu M, Wang C, Swerdloff R, Lee P (2011). Reexamination of testosterone, dihydrotestosterone, estradiol and estrone levels across the menstrual cycle and in postmenopausal women measured by liquid chromatography–tandem mass spectrometry. Steroids.

[19] Huck UW, Seger J, Lisk RD (1990). Litter sex ratios in the golden hamster vary with time of mating and litter size and are not binomially distributed. Behav Ecol Sociobiol.

[20] Biggers J, Curnow R, Finn C, McLAREN A (1963). Regulation of the gestation period in mice. Reproduction.

[21] Kai O, Sonoda Y, Sensui N, IMAI K (1999). Effects of litter size on gestation length and plasma progesterone concentration in Mongolian gerbils (Meriones unguiculatus). Nihon Chikusan Gakkaiho.

[22] Sensui N, Mizuno H (1980). Effect of reversed lighting regime on the timing of parturition in rats whose litter sizes were surgically adjusted. Japanese Journal of Animal Reproduction.

[23] Hawk H (1983). Sperm survival and transport in the female reproductive tract. J Dairy Sci.

[24] Hull E, Dominguez J, Muschamp J, Lathja A, Blaustein JD (2007). Neurochemistry of male sexual behavior. Handbook of Neurochemistry and Molecular Neurobiology.

[25] Kuehn RE, Zucker I (1968). Reproductive behavior of the Mongolian gerbil (Meriones unguiculatus). Journal of Comparative and Physiological Psychology.

[26] Clark MM, Galef BG (1977). The role of the physical rearing environment in the domestication of the Mongolian gerbil (Meriones unguiculatus). Anim Behav.

[27] Dewsbury DA (1990). Modes of estrus induction as a factor in studies of the reproductive behavior of rodents. Neurosci Biobehav Rev.

[28] Hartung TG, Dewsbury DA (1978). A comparative analysis of copulatory plugs in muroid rodents and their relationship to copulatory behavior. J Mammal.

[29] Peitz B, Foreman D, Schmitt M (1979). The reproductive tract of the male spiny mouse (Acomys cahirinus) and coagulation studies with other species. J Reprod Fertil.

[30] Stockley P, Franco C, Claydon AJ, Davidson A, Hammond DE, Brownridge PJ (2020). Revealing mechanisms of mating plug function under sexual selection. Proc Natl Acad Sci.

[31] Dewsbury DA (1972). Patterns of copulatory behavior in male mammals. Q Rev Biol.

[32] Wang C, Guerriero LE, Huffman DM, Ajwad AA, Brooks TC, Sunderam S (2020). A comparative study of sleep and diurnal patterns in house mouse (Mus musculus) and Spiny mouse (Acomys cahirinus). Sci Rep.

