# Supplementary figures and images for: Good things come to those who mate: analysis of the mating behaviour in the menstruating rodent, Acomys cahirinus
Source: BMC Zool. 2022 Feb 25;7:13. doi: 10.1186/s40850-022-00112-1 (PMC10127372; doi:10.1186/s40850-022-00112-1)

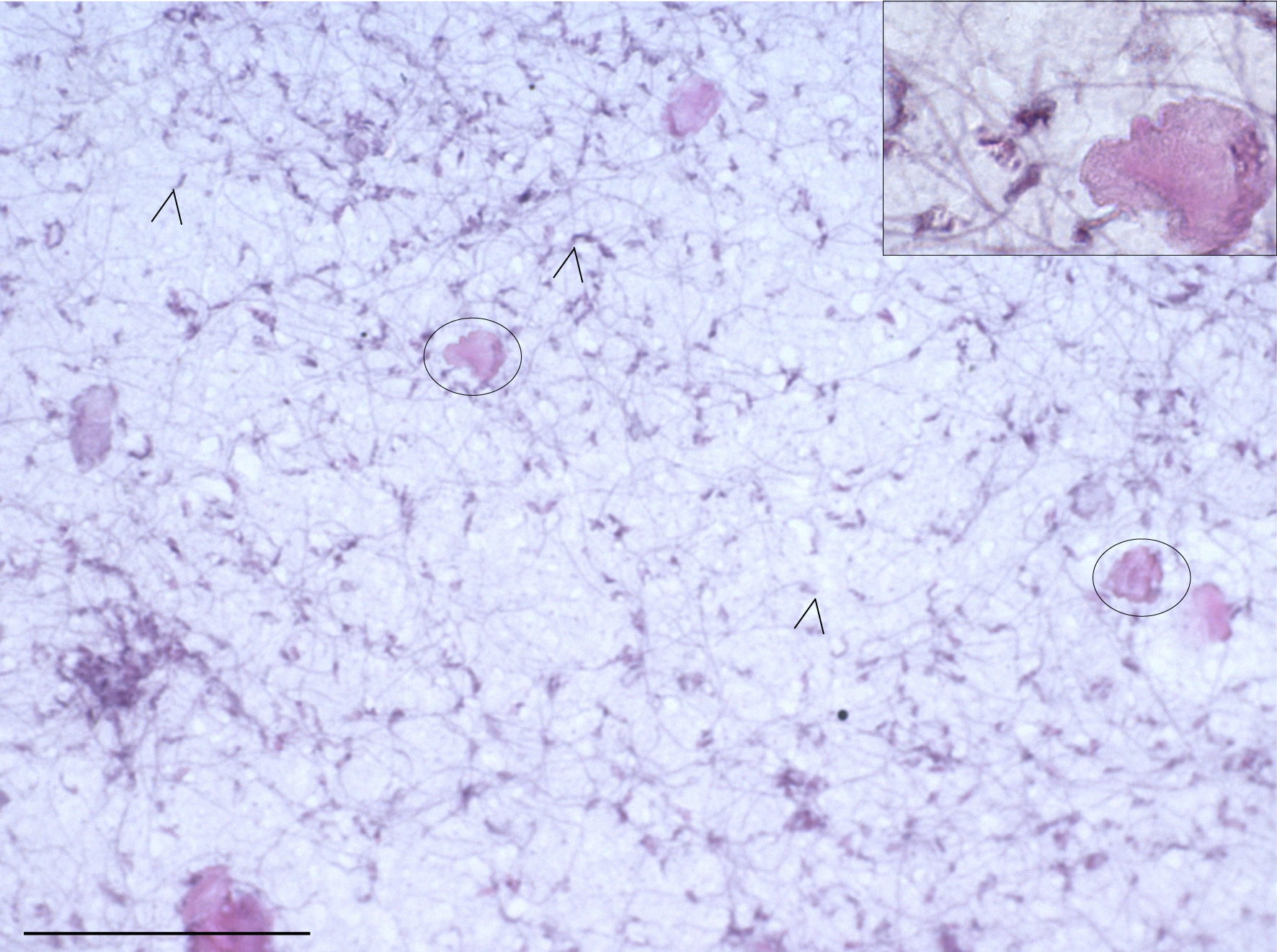

Supplement: Supplementary file 1 — Additional file 1: Supplementary figure 1. Spermatozoa in a vaginal lavage sample following a locking event. Spermatozoa (arrows) and cornified epithelial cells (circles) in the vaginal lavage from a female spiny mouse immediately following an ejaculatory locking event. Scale bar = 100um (40X) and inset square shows spermatozoa and cornified epithelial cells at higher magnification (100X). [file 40850_2022_112_MOESM1_ESM.tif]
